# Gastric carcinosarcoma with rhabdomyosarcomatous differentiation: a case report and review

**DOI:** 10.1186/s40792-016-0176-z

**Published:** 2016-06-02

**Authors:** Masashi Fujiie, Manabu Yamamoto, Kenichi Taguchi, Ayako Iwanaga, Kippei Ohgaki, Akinori Egashira, Kazuhito Minami, Yasushi Toh, Yoshinao Oda, Takeshi Okamura

**Affiliations:** Department of Gastroenterological Surgery, National Kyushu Cancer Center, 3-1-1 Notame, Minami-ku, Fukuoka 811-1395 Japan; Department of Pathology, National Kyushu Cancer Center, 3-1-1 Notame, Minami-ku, Fukuoka 811-1395 Japan; Department of Pathology II, Kyushu University, 3-1-1 Maedashi, Higashi-ku, Fukuoka 812-8582 Japan

**Keywords:** Gastric carcinosarcoma, Rhabdomyosarcomatous, Neuroendocrine

## Abstract

We report an unusual case of gastric carcinosarcoma with rhabdomyosarcomatous and neudoendocrinal differentiation in a 71-year-old Japanese female. Gastric carcinosarcoma with rhabdomyosarcomatous and neuroendocrinal differentiation is a rare tumor. The tumor developed in the body of the stomach and was exophytic in appearance. By histochemical analysis, the tumor was shown a part of positive for desmin and myoglobin and a part of positive for synaphtophysin and vimentin.

We conclude that, though rare, gastric carcinosarcoma with rhabdomyosarcomatous and neuroendocrinal differentiation thus is reviewed in the English literatures.

## Background

Carcinosarcomas are rare, malignant, biphasic tumors. In the upper gastrointestinal tract, they are most frequently observed in the esophagus, while localization in the stomach has been less frequently reported [1–3]. We present a case of a 71-year-old female having gastric carcinosarcoma with rhabdomyosarcomatous and neudoendocrinal differentiation. Rhabdomyosarcomatous differentiation of the stomach is a rare neoplasm with only twelve cases previously reported [4–13].

We reviewed the scientific literature pertaining to gastric rhabdomyosarcoma and identified several distinctive clinical features of this type of tumor.

## Case presentation

**Fig. 1:**
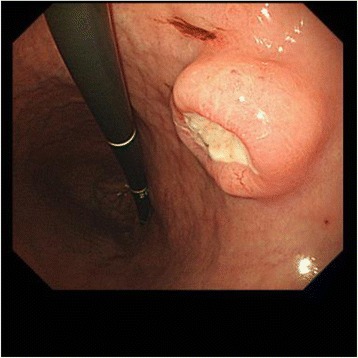
Endoscopy showed an ulcerative lesion in the gastric body

A 71-year-old Japanese female was admitted to the National Kyushu Cancer Center in April, 2012. Anorexia and vomiting were not observed; the CEA and CA19-9 levels were below the cutoff levels. Endoscopic studies revealed a Bormann II type lesion in the middle stomach (Fig. 1); rhabdomyosarcoma was confirmed on biopsy. We performed a laparoscopic distal gastrectomy with D2 dissected lymph node on May, 2012. On macroscopic examination, a 2.0 × 1.5 cm tumoral mass was identified in the body. The tumor invaded up until the subserosa, but no lymph node metastasis was found. As a result, the operation was considered to be curative. The patient was discharged on the 14th postoperative day. She was not admitted adjuvant chemotherapy by her offer. The patient has been doing well without any recurrence for 3 years.

**Fig. 2:**
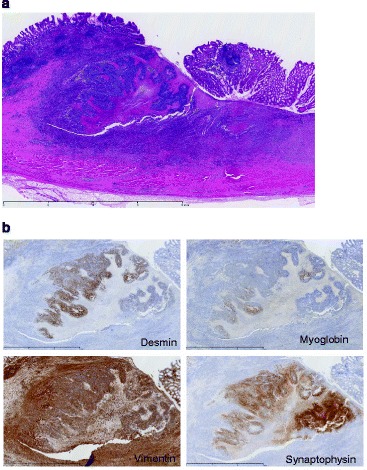
**a** Hematoxylin-stained tumor specimen. **b** Immunohistochemical staining for desmin, myoglobin, vimentin, and synaptophysin was positive

Pathologically, the tumor was identified as carcinosarcoma with skeletal muscle and neuroendocrinal differentiation. In the submucosa, there was a proliferation of oval to polygonal cells with hyperchromatic nuclei, prominent nucleoli, and a small amount of eosinophilic cytoplasm, arranged in sheets and accompanied by thin fibro-vascular septa and prominent necrosis. Mitotic figures were frequently seen. Aggregates of histiocytes and granulation tissue were recognized in the surrounding gastric wall (Fig. 2a).

Immunohistochemical staining was performed using the ordinary avidine-biotin-peroxidase complex, and reaction products were visualized by benzidine reaction. The sections were slightly counterstained by hematoxylin. Immunohistochemically, the tumor cells were positive for AE1/AE3, synaptophysin, desmin, and myogenin (Fig. 2b) but negative for CAM5.2, chromogranin A, S-100 protein, c-kit, and DOG1. PAX3/7-FKHR or EWS-WT1 fusion gene transcripts were not detected by RT-PCR [14, 15].

### Discussion

In the Japanese and English scientific literature, about 60 cases of gastric carcinosarcoma have been reported. The most commonly reported carcinomatous component of this tumor is tubular or papillary carcinoma. The sarcomatous components have still not been adequately characterized in most of the reported cases. Distinct sarcomatous components showing heterologous differentiation such as leiomyosarcoma [4, 5, 16–19], rhabdomyosarcoma [4–13, 20], osteosarcoma [5, 9, 21], and chondrosarcoma [5, 22–24] have been reported. In our patient, not only rhabdomyoblastic differentiation but also neuroendocrine features were observed. This association of rhabdomyosarcomatous and neuroendocrinal differentiation has been described in only five cases of carcinosarcoma, affecting the pancreas [25], larynx [26], ileum [27], nanorectal junction [28], and stomach [12]. This case is the second report on gastric carcinosarcoma with rhabdomyosarcomatous and neuroendocrinal differentiation.

There is no indication that the gastric rhabdomyomatous component of gastric carcinosarcoma in our case or in other case reports represented a from some other site; indeed, metastases to the stomach by rhabdomyosarcomas are uncommon. De la Monte et al. reported that no instances of metastasis to the stomach in a review of 17 autopsies of 22 patients who died of embryonal and alveolar rhabdomyosarcoma at Johns Hopkins Hospital between 1929 and 1983 [29].

Gastric carcinosarcoma with rhabdomyosarcomatous differentiation has been reported in twelve cases (Table 1) [4–13]. In these twelve cases, no clinical feature has been associated with age, sex, or location. However, most cases showed a polypoid lesion, and in three of the 12 cases, it was recognized in the remnant stomach. There are cases which had poor prognosis. Actually, three of eight cases, which could confirm their survivals, were died within 1 year. The tendency of gastric rhabdomyosarcoma to metastasize to lymph node and lungs is in agreement with previous observations of rhabdomyosarcoma emerging at other sites [30].

**Table 1:** Reported cases of gastric carcinosarcoma with rhabdomyosarcomatous differentiation

Author	Age/sex	Location	Size (cm)	Gross feature	Depth of invasion		Other specified histology	Outcome
					carcinoma	sarcoma		
Stout (1953) [8]					Submucosa			
Stout (1953) [8]					Submucosa			
Kyogoku (1960) [4]	49/M						Leiomyosarcoma Fibrosarcoma	Dead, 36 mon
Matsukuma (1977) [9]	74/M	Remnant S	15 × 11	Polypoid	Serosa	Muscularis propria	Osteosarcoma Fibrosarcoma	Dead, 5 mon (Liver meta)
Machida (1981) [7]	39/F	Cardia	7.0 × 6.0	Polypoid	Submucosa	Submucosa	Chondrosarcoma Leiomyosarcoma	Dead, 5mon
Fox (1990) [10]	68/F	Body	15 × 10	Polypoid	Mucosa			Dead, 26 mon
Sugai (1991) [11]	78/M	Pylorus	9.0 × 7.0	Polypoid	Serosa		Spindle cell sarcoma	Dead, 5 mon
Melato (1993) [5]	73/M	Remnant S	7.0 × 5.5	Polypoid	Serosa	Serosa	Fibrosarcoma Chondrosarcoma	
Nakayama(1997) [6]	69/M	Remnant S	20 × 18	Polypoid			Osteoblastic	Autopsy (Pheumonia)
Tsuneyama(1999) [12]	63/M	Pylorus	7.0 × 6.5	Polypoid	Subserosa		Neuroendocrine tumor	Alive 10 mon
Sato Y (2001) [13]	67/F	Fundus	8.0 × 7.0	Polypoid			Adenosquamous ca	Alive 11 mon
Surg Case report 2016 (In press)	71/F	Body	2.0 × 1.5	Polypoid	Subserosa	Subserosa	Neuroendocrine tumor	Alive, 36 mon

*Remnant S* Remnant stomach

The histogenesis of gastric carcinosarcoma remains controversial. Some authors have reported a bioclonal origin, which supports the collision tumor theory [16, 31]. Others have proposed that these tumors are monoclonal and that the sarcomatous elements originate from a common stem cell that has the ability to undergo both epithelial and mesenchymal differentiation [7, 32]. In our patient, there were occasional transitions between carcinomatous and sarcomatous components, and the immunohistochemical detection of stem in sarcomatous cells may suggest the sarcomatous differentiation of adenocarcinoma. During transdifferentiation, the occurrence of stem cells with multi-differentiation ability is capable to explain the variety of cell types observed in the present tumor.

Our experience with the present case emphasizes that gastric carcinosarcoma with rhabdomyosarcomatous differentiation exhibits aggressive behavior, the tumor, however, is extremely rare.

## Conclusions

This report described a very rare case of gastric carcinosarcoma with rhabdomyosarcomatous lesions. This case has survived without tumor recurrence, though most cases of gastric carcinosarcoma with rhabdomyosarcomatous were poor prognosis.

## Consent for publication

Patient consent for publication of images has been given in writing.
